# Comparing DXA and BIA for Assessing Changes in Muscle Mass in Older Adults Following Strength Training

**DOI:** 10.1093/geroni/igaf122.3903

**Published:** 2025-12-31

**Authors:** Daniel Lee, Afeo Ikhide, Noberto Quiles, Bridget McFadden, Anoop Balachandran

**Affiliations:** Queens College, Flushing, New York, United States; Queens College, Flushing, New York, United States; Queens College, Flushing, New York, United States; Queens College, Flushing, New York, United States; Queens College, Flushing, New York, United States

## Abstract

Dual-energy X-ray absorptiometry (DXA) is frequently used to assess sarcopenia and muscle mass in older adults. However, DXA is expensive, requires a licensed X-ray technician, and involves radiation exposure. In contrast, bioelectrical impedance analysis (BIA) is quick, cheaper, requires no expertise, and does not involve radiation. Despite these practical advantages, comparative studies investigating change in fat-free mass (FFM) in response to resistance training in healthy older adults are limited. We compared DXA vs. BIA for FFM assessment in response to a strength training program in older adults. Community-dwelling older adults (n = 11, M = 68 years [SD 3.3], 75% female) underwent both DXA and BIA measurements under standardized conditions at baseline and after 20 weeks. Full-body strength training regimen of eight exercises was performed twice a week for 20 weeks using plate-loaded machines. The intraclass correlation coefficient (ICC) was used to assess absolute agreement. Bland–Altman (BA) plots were used to evaluate measurement error and limits of agreement. Absolute agreement for change in FFM between DXA and BIA was good, with an ICC of 0.86 (95% CI [.56, .96]), F(10,10) = 12.38, p < .001. The BA analysis showed a mean difference of 0.04 kg (95% CI [−0.35, 0.43]), with limits of agreement ranging from −1.09 to 1.17 kg. Our findings demonstrate good agreement between DXA and BIA for change in FFM in response to strength training in older adults. However, a larger sample size is needed to draw stronger conclusions about the utility of BIA.

